# Anxiety, Depression, and Concern About Employment Status of Hotel Housekeepers in the Balearic Islands During the COVID-19 Pandemic: A Longitudinal Study

**DOI:** 10.3389/fpsyg.2022.842335

**Published:** 2022-04-05

**Authors:** Xenia Chela-Alvarez, Alfonso Leiva, Laura Gallardo-Alfaro, Oana Bulilete, MClara Vidal-Thomas, Joan Llobera

**Affiliations:** ^1^Primary Care Research Unit of Mallorca, Balearic Islands Health Service, Palma, Spain; ^2^GrAPP-caIB – Health Research Institute of the Balearic Islands (IdISBa), Palma, Spain; ^3^RICAPPS- Red de Investigación Cooperativa de Atención Primaria y Promoción de la Salud – Carlos III Health Institute (ISCIII), Madrid, Spain

**Keywords:** anxiety, depression, employment status, COVID-19, hotel housekeepers

## Abstract

Tourism is a crucial economic sector in the Balearic Islands (Spain). COVID-19 pandemic might severely impact hotel housekeepers (HHs) due to their already precarious employment situation. The purpose is to assess the evolution of the concern about employment status, anxiety, and depression of HHs. This is a longitudinal study conducted with a subset of participants from a primary care, health promotion intervention study. Two additional visits were added (March–April and October–December 2020) for the purpose of this study. We recruited 290 HHs in March–April 2020; 237 were again interviewed during October–December 2020. In the first visit, high level of concern about employment was associated with age under 50, temporary contracts and external locus of control (LOC). Moderate–severe anxiety was associated with low social support and external LOC; moderate–severe depression was associated with low social support. Regarding the second visit, age, years working as HH, type of contract, social support, and LOC were not associated with concern about employment status, anxiety, and depression. There was a larger proportion of HHs with moderate–severe anxiety and depression among HHs with high degree of concern. Concern increased significantly among HHs: over 50 years of age; with more than 15 years in the job, a recurring seasonal contract and normal social support. After adjusting by age, type of contract, LOC, and social support, we found a statistically significant increase (12.0%) of HHs highly concerned about their job situation: compared to the first visit, HHs were 2.3 more likely to have a high degree of concern in December 2020. In contrast, increases in moderate–severe anxiety (0.3%) and depression (4.3%) between the two periods were not significant. In HHs, the COVID-19 pandemic has caused significant concern about employment status and symptoms of depression and anxiety. In the uncertain times of the pandemic, mental wellbeing benefits from variables that confer stability, such as internal LOC, perception of social support, and a stable job. Longitudinal results point at long lasting effects of the COVID-19 pandemic on mental health. It is crucial to allocate additional resources in primary care to adequately address the anticipated influx of needs.

## Introduction

The first diagnosed case of COVID-19 in Europe occurred in mid-January of 2020. The virus quickly spread throughout the continent, forcing governments to apply stringent control measures that negatively impacted the daily life of citizens, such as home confinement and closure of schools and establishments. In Spain, the lockdown officially began on 14 March 2020. While the COVID-19 pandemic had recognizable effects on the physical health of infected individuals, it also adversely affected the mental health of general population (Gao et al., 2020; Huang and Zhao, 2020; Mazza et al., 2020; Ozamiz-Etxebarria et al., 2020; Wang et al., 2020a; Zhang et al., 2020; O’Connor et al., 2021).

The measures to control COVID-19 had a drastic impact on the Spanish tourism industry. In 2018, tourism accounted for 44% of the gross domestic product (GDP) in the Balearic Islands. The Social and Economic Council of the Balearic Islands forecasted a decline of 75 to 80% in the hospitality sector by the end of 2020 (Consell Econòmic I Social de les Illes Balears, 2020). Predictably, the number of tourists arriving in the Balearic Islands fell from 16.4 million in 2019 to 3.1 in 2020 (Balearic Islands Statistics Institute). The number of tourists in Spain also fell from 83.5 M in 2019 to 18.9 M in 2020, and the contribution of the tourism sector to the GDP in 2020 was 61.4 billion Euros, less than half that of the previous year (Instituto Nacional de Estadística, 2020).

An estimated 13,000 hotel housekeepers (HHs) work in the Balearic Islands, a female-dominated sector in Spain (practically 100%; Cañada, 2018). Most HHs had recurring seasonal or temporary contracts, with no guaranteed income throughout the year. This precarious occupational status was expected to worsen. Some international organizations had forecasted that women would be more vulnerable to the COVID-19 economic crisis and affected for longer due to gender inequalities in a wide range of dimensions (income, wealth, type of jobs, and other; European Institute for Gender Equality, 2020; Organisation for Economic Cooperation and Development, 2020). Moreover, the hospitality sector has been identified as particularly sensitive to temporary layoffs (European Institute for Gender Equality, 2020). Consequently, we anticipated a major negative impact of the current socioeconomic crisis on this occupational group.

To date, several studies have examined the psychological impact of COVID-19 on the general population. Most studies recruited the sample through social networks and enrolled high percentages of well-educated people. The results of these works emphasized higher prevalences of anxiety and depression (Gao et al., 2020; Huang and Zhao, 2020), especially in women and young people (Hidalgo et al., 2020; Huang and Zhao, 2020; Mazza et al., 2020; Nienhuis and Lesser, 2020; Ozamiz-Etxebarria et al., 2020; Wang et al., 2020a; Valiente et al., 2021). In Spain, among women, prevalence of chronic anxiety was 9.1% and the prevalence of chronic depression was 9.2% in 2017 (Sanidad, 2018); in the first stages of the Spanish lockdown, Jacques-Aviñó et al. (2020) found that 31.2% of women reported anxiety symptoms and 28.5%, depression symptoms. Some studies reported poorer physical and mental health status among people who experienced work-related changes (i.e., stopped working, lost their job, and reduced working hours) during the COVID-19 pandemic (Nienhuis and Lesser, 2020; Rodríguez-Rey et al., 2020; Zhang et al., 2020). More women than men reported work-related changes (i.e., reduced hours and loss of employment), and these women expressed more generalized anxiety symptoms compared to women who had not experienced these changes (Nienhuis and Lesser, 2020). In addition, income level showed a negative correlation with fear of infection, disease and death, lack of basic necessities, social isolation, loss of work, and income (Sandín et al., 2020). During COVID-19 pandemic, lower income has been directly associated with increased anxiety and depressive symptoms (Fancourt et al., 2021; Hertz-Palmor et al., 2021).

Some longitudinal studies have analyzed population mental wellbeing throughout lockdown and afterward. Results are conflicting, since while some authors did not find significant changes in depression (Wang et al., 2020b; O’Connor et al., 2021; Pieh et al., 2021; Somma et al., 2021) and anxiety (González-Sanguino et al., 2021; Pieh et al., 2021), others show significant changes in depression and/or anxiety levels (Salfi et al., 2020; Fancourt et al., 2021; González-Sanguino et al., 2021; O’Connor et al., 2021; Somma et al., 2021). Overall, most evidence suggests an improvement in mental health indicators (or at least not deterioration) from the first stages of lockdown to weeks and months later. However, data on mental health among workers outside the health sector are scarce (Savolainen et al., 2021).

Lockdown also entailed an increase in household and childcare tasks for both men and women given the school closures and the impossibility to outsource housework. Evidence has shown that, in several countries—including Spain—, despite the increase of men’s participation in housework and childcare, most of the burden continued falling on women (Giurge et al., 2021; Yaish et al., 2021), irrespective the status in the labor market (Farré et al., 2021). Additionally, Farré et al. (2021) showed that the decreasing in paid work during the lockdown affected more men than women, resulting in women working longer hours. Thus, the crisis appears to have widened the gender gap in both paid an unpaid work.

This is a longitudinal study focused on HHs, a female workforce characterized by precarious employment in a sector specially affected by COVID-19 control measures. Due to the sociodemographic characteristics of our sample and the extended restrictions to tourism, we anticipate that HHs will be a population group strongly affected by the pandemic. We hypothesized that HHs’ concerns for employment status, anxiety, and depression symptoms will increased from 1st visit: March–April 2020 to the 2nd visit: October–December 2020, and this increased could be related to age, more precarious employment status (temporary or recurring seasonal contract), years working as HHs, low social support, and an external LOC.

The objective of this study is to evaluate the degree of concern on employment status, anxiety, and depression symptoms of the first 6 months of the COVID-19 pandemic on HHs: firstly, during the first stages of lockdown and secondly, in October–December 2020. In the last trimester 2020, Spain was in the second wave of COVID-19; consequently, on the 25th October, Spanish government ruled a new state of alarm and autonomous communities adopted appropriate measures adapted to their pandemic situation. In autonomous community of the Balearic Islands, during the last trimester 2020, there were limitations in the capacity of indoor places, as well as limitations of number of people in the meetings, and curfew between 12 pm and 6 pm. We evaluated the concerns of HHs regarding their employment status and also their psychological wellbeing. The results of this study should alert occupational health services and prompt measures to improve the wellbeing of HHs.

## Materials and Methods

The study was conducted by the Primary Care Research Unit of Mallorca. Participants were a selected subset from a randomized controlled, parallel, and multicenter intervention trial to assess the efficacy of a complex intervention conducted in the primary healthcare setting, based on behavioral changes to improve the quality of life of HHs.^1^ We added two visits (the first in March–April and the second in October–December 2020) to collect data for this study.

### Study Population and Recruitment

Eligibility criteria age over 18 years; having health coverage in the Balearic Public Health System; having worked as HH during 2019; and consent to participate. Exclusion criteria were language barrier (not understanding Spanish or Catalan); pregnancy; being on sick leave; participating in another clinical trial aiming at modifying life styles; severe psychiatric illness; and any positive answer to the physical activity aptitude questionnaire proposed by the American College of Sports Medicine (Adams, 1999). A minimum of 269 subjects were needed to detect an increase (between the start of the pandemic and after 6 months) of at least 8% in the proportion of HHs with moderate–severe depression symptoms (measured by the PHQ-9) with 80% power, a two-sided α value of 0.05, and assuming 20% of loss to follow-up.

### Instruments

The locus of control (LOC), a measure of coping capacity defined as the perception about the cause of events happening in your life, was assessed by answering the question: To what extent do you agree with the statement: “I feel that what happens in my life is often determined by factors beyond my control?” Answers were rated on a 6-point Likert scale (Mounce et al., 2018). LOC was classified as “internal” or “external.” A person with an internal LOC believes that what happens in life is a consequence of one’s own actions, attitudes, or behaviors, whereas a person with an external LOC believes that life events are the result of fortune, fate, or the actions of others (Rotter, 1966).

The Duke-UNC-11 (Broadhead et al., 1988), an 11-item questionnaire that measured functional elements of social support (including confidant and affective support) and validated for the Spanish population (Cronbach’s Alpha = 0.90; de la Revilla Ahumada et al., 1991; Bellón Saameño et al., 1996), was used to measure social support. Each item is valued in a 5-points scale ranging from 1 (“much less than I would like”) to 5 points (“as much as I would like”). A final score ranging from 5 to 55 is obtained; 32 points or below correspond to a low social support; and more than 32 points correspond to a normal social support (Bellón Saameño et al., 1996).

Mental health status was assessed using the Generalized Anxiety Disorder (GAD-7; Spitzer et al., 2006) in its Spanish validated version (García-Campayo et al., 2010), a 7-item questionnaire that assesses self-perceived anxiety during the previous 2 weeks (Cronbach’s Alpha = 0.936). For each question, the respondent gives an answer of 0 (not at all), 1 (several days), 2 (more than half of the days), or 3 (nearly every day). The Patient Health Questionnaire (PHQ-9; Spitzer et al., 2000; Diez-Quevedo et al., 2001) is a 9-item questionnaire (Cohen’s Kappa = 0.74), with an additional question that measures depressive disorders during the previous 2 weeks. As above, for each question, the respondent gives an answer of 0 (not at all), 1 (several days), 2 (more than half of the days), or 3 (nearly every day).

Concern about employment status was asked with the question “In a scale from 1 to 5, in which 1 is ‘the lowest degree of concern’ and 5 ‘the highest degree of concern’, to what extent are you worried about your employment status after lockdown?”

### Procedures

In the first visit (March 25–April 17, 2020), we recorded the following sociodemographic variables: age, nationality, education level, years working as HH, type of contract, and employment status. Since 14 March 2020, all participants were under mandatory lockdown (home confinement, only leaving the house for essential activities, such as going to the supermarket, or the pharmacy). The second visit was done between October and December 2020, 4 months after the end of the lockdown. In both visits, we recorded LOC, social support (DUKE-UNC-11), anxiety (GAD-7), depression (PHQ-9), and degree of concern for employment status.

### Data Analysis

Demographic and occupational characteristics, employment status, locus of control, social support, degree of concern for employment status, anxiety, and depression outcomes are shown with absolute numbers and percentages. Age and years working as HH are presented as means and standard deviation (SD). To compare demographic and occupational characteristics, employment status, LOC, social support, degree of concern for employment status, anxiety (GAD7), and depression (PHQ-9), we used the chi-squared test; values of *p* under 0.05 were considered statistically significant (two-sided tests). A limit of *p* < 0.20 was used to select predictors in the multivariate analysis. Repeated measure Generalized Estimating Equations (GEE) were used to estimate the odds ratios (OR) and 95% confidence interval (CI) of the changes over time in anxiety, depression, and concern about employment status associated to demographic and occupational characteristics, employment status, LOC, and social support of the participants (Liang and Zeger, 1986: 22) and Cohen’s h index; the arcsine transformation of the proportion of patients with high degree of concern about employment status, moderate or severe anxiety (GAD7), or depression (PHQ) between the two study periods was calculated. McNemar’s test was used for the association between independent variables and the evolution of concern for employment status and mental wellbeing.

Missing outcomes were accounted for using multiple imputation with chained equation (White et al., 2011). Ten imputed samples were generated and estimates were combined using Rubin’s rules (Rubin, 2004).

All statistical analyses were done with SPSS for Windows version 23.0.

### Ethical Consideration

All HHs included in this study were already participating in an approved RCT.^2^ The study was approved by the Balearic Islands Research Ethics Committee (IB 4187/20 PI).

## Results

A total of 290 HHs were recruited from 25 March to 17 April 2020, and 237 (82%) were again interviewed for a second visit in October–December of 2020. Participants’ characteristics are displayed in Table 1 and include as: individual, sociodemographic, and working variables. Most HHs included had completed compulsory education (primary and secondary), were Spanish nationals, and had a recurring seasonal contract. Before lockdown, half of the sample was receiving unemployment benefits.

**Table 1:** Characteristics of the study population.

	**Hotel housekeepers (*n* = 290)**
	**x̄ (SD)**
Age	46.8 (±10.2)
Years working as HHs	14.3 (±10)
	**% (95%CI)**
**Education level**	
Primary not finished	3.6 (1.8–6.6)
Compulsory education	63.4 (57.4–69.1)
Secondary post-compulsory education	26.4 (21.3–32.1)
Higher education	6.5 (3.9–10.1)
**Nationality**	
Spanish	65.8 (60.0–71.6)
Foreign	19.3 (14.4–24.1)
Dual	14.9 (10.5–19.3)
**Origin**	
Spain	64.7 (58.8–70.4)
Africa	5.5 (3.1–8.8)
America	25.5 (20.4–31.0)
Asia	0.4 (0.0–2.0)
Non-EU	0.4 (0.0–2.0)
EU	3.6 (1.8–6.6)
**Type of contract**	
Permanent	2.5% (0.1–5.1)
Recurring seasonal contract	72.6% (67.0–77.7)
Temporary	24.9% (20.0–30.4)
**Employment status before COVID-19 lockdown**	
Working	28.6% (23.5–34.2)
Unemployment benefits	51.7% (45.8–57.6)
Unemployed	9.0% (5.9–12.9)
Other	10.7% (7.4–14.8)
**LOC**	
Internal	35.4% (29.7–41.5)
External	64.6% (58.5–70.3)
**Social support perceived (DUKE-UNC-11)**	
Low	5.4% (3.0–8.8)
Normal	94.6% (91.2–97.0)

The Spanish lockdown began on 14 March and finished on 21 June. During this period, the HHs showed a high degree of concern about their job, with 15 and 21% presenting high levels of anxiety (GAD7 Cronbach’s Alpha = 0.874) and depression (PHQ9 Cronbach’s Alpha = 0.875), respectively. We analyzed the association between age, years working as HH, type of contract, social support, and LOC, with the degree of concern for employment status and mental wellbeing (anxiety and depression) for the first and the second visits. In the first visit, more concern about employment status was reported in women under 50 years of age (*p* = 0.014), with a temporary contract (*p* = 0.049) and with an external LOC (*p* = 0.017). Regarding moderate–severe anxiety, a positive significant association was found in cases with low social support (*p* = 0.018) and external LOC (*p* = 0.049); moderate–severe depression was significantly related to low social support (*p* = 0.001). Interestingly, data of the second visit failed to indicate any association between baseline values and concern about employment status, anxiety, and depression.

Table 2 shows the comparison of concern about employment status, anxiety, and depression between the two periods (1st visit: March–April 2020; 2nd visit: October–December 2020) by age, years of working as HH, type of contract, social support, and LOC. A statistically significant increase from the 1st to the 2nd visit in the concern about employment status was detected in HHs: over 50 years of age (1st visit: 66.9%; 2nd visit: 92.0%; *p* < 0.001); who had worked as HH more than 15 years (1st visit: 70.9%; 2nd visit: 85.9%; *p* = 0.007); with a recurring seasonal contract (1st visit: 68.9%; 2nd visit: 83.1%; *p* = 0.002); with normal social support (1st visit: 72.5%; 2nd visit: 86.5%; *p* < 0.001). Regarding LOC, there was an increase in the degree of concern without differences between internal (*p* = 0.017) and external LOC (*p* = 0.008).

**Table 2:** Association between hotel housekeepers characteristics (age, years working as HHs, type of contract, social support, and LOC) and degree of concern about employment status and mental wellbeing.

		Degree of concern about employment status					GAD7					PHQ9				
		1st visit		2nd visit		value of *p*	1st visit		2nd visit		value of p	1st visit		2nd visit		value of *p*
		Low n/N (%)	High n/N (%)	Low n/N (%)	High n/N (%)		Mild n/N (%)	Moderate–severe n/N (%)	Mild n/N (%)	Moderate–severe n/N (%)		Minimum-Mild n/N (%)	Moderate–severe n/N (%)	Minimum-Mild n/N (%)	Moderate–severe n/N (%)	
Age	<40	15/72 (20.8)	57/72 (79.2)	10/55 (18.2)	45/55 (81.8)	1.000	49/72 (68.1)	23/72 (31.9)	38/54 (70.4)	16/54 (29.6)	0.804	57/72 (79.2)	15/72 (20.8)	43/52 (82.7)	9/52 (17.3)	0.774
	41–50 years	12/77 (15.6)	65/77 (84.4)	11/67 (16.4)	56/67 (84.6)	1.000	63/77 (81.8)	14/77 (18.2)	42/61 (68.9)	19/61 (31.1)	0.238	60/77 (77.9)	17/77 (22.1)	50/62 (80.6)	12/62 (19.4)	1.000
	>50	40/121 (33.1)	81/121 (66.9)	9/97 (9.3)	88/97 (90.7)	<0.001	92/121 (76.0)	29/121 (24.0)	80/101 (79.2)	21/101 (20.8)	0.359	95/121 (78.5)	26/121 (21.5)	83/100 (83.0)	17/100 (17.0)	0.118
Years working as HHs	≤5	16/67 (23.9)	51/67 (76.1)	8/53 (15.1)	45/53 (84.9)	0.289	52/67 (77.6)	15/67 (22.4)	42/50 (84.0)	8/50 (16.0)	0.180	54/67 (80.6)	13/67 (19.4)	41/47 (87.2)	6/47 (12.8)	0.727
	6–15 years	27/106 (25.5)	79/106 (74.5)	10/85 (11.8)	75/85 (88.2)	0.052	78/106 (73.6)	28/106 (26.4)	59/82 (72.0)	23/82 (28.0)	1.000	77/106 (72.6)	29/106 (27.4)	67/80 (83.8)	13/80 (16.3)	0.134
	>15	30/103 (29.1)	73/103 (70.9)	12/84 (14.3)	72/84 (85.7)	0.007	79/103 (76.7)	24/103 (23.3)	65/89 (73.0)	24/89 (27.0)	0.832	84/103 (81.6)	19/103 (18.4)	70/89 (78.7)	19/89 (21.3)	1.000
Type of con-tract	Permanent	2/7 (28.6)	5/7 (71.4)	0/5 (0)	5/5 (100)	NA	4/7 (57.1)	3/7 (42.9)	3/5 (60.0)	2/5 (40.0)	1.000	5/7 (71.4)	2/7 (28.6)	4/5 (80.0)	1/5 (20.0)	1.000
	Recurring seasonal	60/204 (29.4)	144/204 (70.6)	27/166 (16.3)	139/166 (83.7)	0.002	157/204 (77.0)	47/204 (23.0)	122/165 (73.9)	43/165 (26.1)	0.880	159/204 (77.9)	45/204 (22.1)	133/162 (82.1)	29/162 (17.9)	0.311
	Temporary	10/69 (14.5)	59/69 (85.5)	4/57 (7.0)	53/57 (93.0)	0.453	53/69 (76.8)	16/69 (23.2)	43/54 (79.6)	11/54 (19.6)	0.508	56/69 (81.2)	13/69 (18.8)	44/53 (83.0)	9/53 (17.0)	1.000
Social support	Low	3/19 (15.8)	16/19 (84.2)	2/17 (11.8)	15/17 (88.2)	1.000	10/19 (52.6)	9/19 (47.4)	10/17 (58.8)	7/17 (41.2)	0.500	9/19 (47.4)	10/19 (52.6)	12/17 (70.6)	5/17 (29.4)	0.125
	Normal	69/251 (27.5)	182/251 (72.5)	27/200 (13.5)	173/200 (86.5)	<0.001	193/251 (76.9)	58/251 (23.1)	154/198 (77.8)	44/198 (22.2)	0.672	200/251 (79.7)	51/251 (20.3)	162/193 (83.9)	31/193 (16.1)	0.349
LOC	External	41/184 (22.3)	143/184 (77.7)	18/155 (11.6)	137/155 (88.4)	0.008	133/184 (72.3)	51/184 (27.7)	108/147 (73.5)	39/147 (26.5)	0.618	141/184 (76.6)	43/184 (23.4)	118/143 (82.5)	25/143 (17.5)	0.185
	Internal	32/89 (36.0)	57/89 (64.0)	12/66 (18.2)	54/66 (81.8)	0.017	74/89 (83.1)	15/89 (16.9)	56/72 (77.8)	16/72 (22.2)	0.815	72/89 (80.9)	17/89 (19.1)	58/71 (81.7)	13/71 (18.3)	1.000

The evolution of anxiety and depression did not differ significantly by age, years working as HH, type of contract, social support, and LOC.

**Figure 1:**
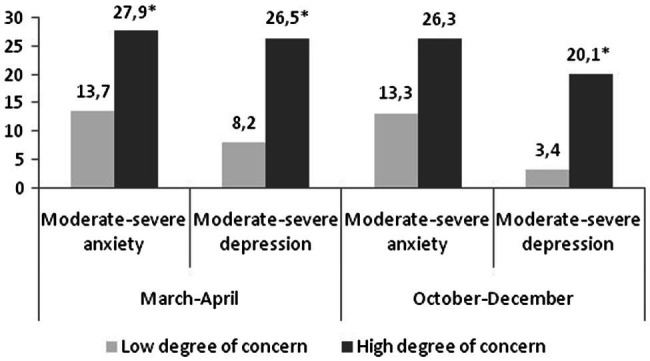
Association between degree of concern for employment status and mental wellbeing.

Figure 1 shows the association between degree of concern and mental wellbeing (moderate–severe anxiety and depression). In March–April, there was a higher proportion of HHs with moderate–severe anxiety (*p* = 0.014) and depression (*p* = 0.001) reporting high degree of concern about their employment status. In October–December, moderate or severe anxiety or depression was more prevalent in HHs with a high degree of concern about employment status. Although these differences were important in magnitude, only reached statistically significance in depression symptoms (*p* = 0.029).

Table 3 shows the evolution of mental wellbeing and degree of concern about employment status from March–April 2020 to October–December 2020; the proportion of HHs declaring a high degree of concern about their employment status (4 and 5 in the Likert scale) increased by 12.0% between the first and the second visit. This increase was statistically significant (adjusted by age, type of contract, LOC, and social support): compared to March–April 2020, HHs were 2.3 more likely to have a high degree of concern for their employment status in October–December. The Cohen’s h effect size was 0.3. HHs with moderate–severe symptoms of anxiety decreased by 0.5% during the period studied (Table 3); this decrease was not statistically significant (adjusted by age, type of contract, LOC, and social support), and the Cohen’s h effect size was 0.012. Although not statistically significant, participants with moderate–severe symptoms of depression decreased by 3.4% from March–April to October–December 2020 (Cohen’s h effect size was 0.086).

**Table 3:** Evolution of high degree of concern about employment status (4 and 5), moderate–severe anxiety, and moderate–severe depression between March–April to October–December 2020.

	First visit (*n* = 237)	Second visit (*n* = 237)	Adjusted OR	95%CI		*p*
	% (95%CI)	% (95%CI)		*LL*	*UL*	
						
High degree of concern about employment status	74.0% (67.9–79.5)	86.0% (80.7–90.3)	2.302	0.400	1.269	>0.001
GAD-7 moderate–severe	25.1%(19.7–31.2)	24.6% (19.2–30.6)	1.016	−0.345	0.377	0.931
PHQ-9 moderate–severe	20.9%(15.8–26.6)	17.5% (12.8–23.1)	0.823	−0.590	0.202	0.337

CI, confidence interval; LL, lower limit; and UL, upper limit.

## Discussion

This longitudinal study started during the first stages of the Spanish COVID-19 lockdown (from March 14 to June 21) and continued in October–December of 2020, when the second wave of COVID-19 was ongoing, lockdown was not current but limitations in the capacity in indoor places and curfew from 12 pm to 6 am were in force. Participants were HHs living in the Balearic Islands, a region economically and socially dominated by tourism. The results reflect the increasing concern of HHs about employment status, probably due to the job insecurity and income loss caused by COVID-19 control measures.

During the Spanish COVID-19 lockdown, HHs showed a high degree of concern about employment status and a high level of anxiety and depression. There was an increase in the degree of concern by employment status from March–April 2020 to October–December 2020 but not in anxiety or depression.

Results showed that HHs who were younger, had temporary jobs and an external LOC were more concerned about their employment status. Low social support and an external LOC were associated to high level of anxiety, and low social support was associated with symptoms of depression. Young age (Rodríguez-Rey et al., 2020; Solomou and Constantinidou, 2020; Ueda et al., 2020; Hubbard et al., 2021), unemployment or job instability (Pieh et al., 2020; Solomou and Constantinidou, 2020; Ueda et al., 2020), external LOC (Sigurvinsdottir et al., 2020), and social support (Hubbard et al., 2021) have been identified as significant to mental wellbeing during the COVID-19 pandemic. Our results support the hypothesis that during the uncertain times of the pandemic, variables conducive to wellbeing are associated with stability, i.e., having an internal LOC, feeling socially supported, and having a stable job. However, the evolution of concern for employment status showed that positive effects of having an internal LOC and a normal social support seemed to have played an important role in the short term. Results indicate that after 6 months, proportions of HHs with high degree of concern for their employment status according to social support and LOC tend to converge.

Results showed a higher prevalence of moderate–severe symptoms of anxiety among HHs compared to Spanish women during the initial stage of the COVID-19 pandemic (Ozamiz-Etxebarria et al., 2020) and compared to Spanish (Rodríguez-Rey et al., 2020) and Italian (Mazza et al., 2020) general populations. HHs also had a higher prevalence of symptoms of depression during this pandemic than Spanish women (Ozamiz-Etxebarria et al., 2020). However, the prevalence of depression was lower than reported by the Spanish (Rodríguez-Rey et al., 2020) and Italian (Mazza et al., 2020) general populations, and similar to that reported by Chinese population (Huang and Zhao, 2020). The divergence in the prevalence of anxiety and depression in different populations might be explained by different participant recruitment procedures, different representativeness of the population samples, or the timing of data collection (early versus later stages of the pandemic). However, these differences can also be explained by the impact of the pandemic on the working population depending on type of employment and sociodemographic and family factors. Most HHs in our sample worked under recurring seasonal contracts, by which HHs just work during the touristic season but the establishment commits to hire them again the following season. Results of the first visit detected a higher degree of concern in a greater proportion of HHs with a temporary contract. Indeed, in March 2020, two-thirds of participants were unemployed, which worsened their already precarious and uncertain occupational status. Other studies have linked employment status to mental wellbeing: in a sample of Japanese workers, symptoms of depression were more prevalent in participants with an unstable job (Ueda et al., 2020).

Additionally, levels of anxiety and depression could be explained by different impacts on geographic regions regarding their social and work environments. We found that 1 out of 4 HHs reported poor psychological wellbeing during the first stages of lockdown, a proportion slightly lower than in European female workers during the 2008 recession (Schütte et al., 2014). Notably, pre-pandemic data collected for the Spanish Health Survey (2017; Sanidad, 2018) show that 9.1% of Spanish women had chronic anxiety and 9.2% chronic depression. These data confirm that anxiety and depression rates have considerably increased as a result of the COVID-19 pandemic. In this context, concern about employment status might play an important role among HHs.

In the participants of this study, concern about employment status increased over time regardless of age, type of contract, LOC, and social support. When interviews were conducted in March–April, the employment uncertainty in hospitality sector was high: HHs were unsure whether they would be able to work during the summer and there were doubts about the governmental Temporary Workforce Adjustment Plan—who would be protected, for how long, how much they would earn, etc. Probably, increase in concern in the second visit reflects the fact that HHs realized that uncertainty about when they would be able to work would last for several months. Our findings underscore the negative association between concern for employment status and mental wellbeing. Accordingly, in Spain, the economic crisis originating from the COVID-19 pandemic was the main concern among the population (Rodríguez-Rey et al., 2020).

These results indicate long lasting effects of the COVID-19 pandemic on mental health and agree with most longitudinal studies in the general population, which have reported similar outcomes or a decreasing trend of anxiety and depression between these periods (Niedzwiedz et al., 2020; Salfi et al., 2020; Fancourt et al., 2021; González-Sanguino et al., 2021; O’Connor et al., 2021; Pieh et al., 2021; Somma et al., 2021). Our results regarding anxiety and depression might be explained by the job uncertainty of HHs. Previous research shows that not working, ceasing to work during lockdown, losing, or perceiving a high risk of losing their job had a significant association with stress, anxiety, and depression (Mazza et al., 2020; Pieh et al., 2020; Rodríguez-Rey et al., 2020; Solomou and Constantinidou, 2020; Ueda et al., 2020; Burstyn and Huynh, 2021). Other factors related to mental distress during the pandemic were being female (Mazza et al., 2020; Pieh et al., 2020; Rodríguez-Rey et al., 2020; Solomou and Constantinidou, 2020; Hubbard et al., 2021), having a lower educational level (Mazza et al., 2020; Rodríguez-Rey et al., 2020; Fancourt et al., 2021) and a low income or financial difficulties (Pieh et al., 2020; Rodríguez-Rey et al., 2020; Ueda et al., 2020; Hubbard et al., 2021; O’Connor et al., 2021).

During the study period, the Spanish government implemented the Temporary Workforce Adjustment Plan, which provided financial assistance to workers with recurring seasonal contracts. We believe that this measure prevented larger increases in anxiety and depression. Nonetheless, our results indicate that the mental health of HHs is still at risk, basically due to the uncertainty related to employment. A greater demand for mental health-related visits in primary care is anticipated. It is essential to allocate additional resources to prevent overwhelming of the primary care services (Roca et al., 2020).

Summarizing, in agreement with the literature, our results indicate that during the pandemic period, there were a high degree of concern about employment status and high rates of anxiety and depression. The results are also a warning about long-term effects on mental health, especially in workers affected by income loss due to enforced governmental measures aimed at controlling the spread of SARS-CoV-2. Importantly, research clearly exposes that the negative impact on mental health is not homogeneously distributed among the population. On the contrary, the people most affected are the most socioeconomically disadvantaged.

### Strengths and Limitations

To our knowledge, many studies on psychological distress caused by the pandemic recruited participants from social networks and most of them well-educated, which do not fully represent the population at large. However, the most studied occupational group was healthcare personnel, overworked and very specifically affected by the pandemic. The relevance of our study lies in focusing attention to the effects of the pandemic on workers’ mental health in economic sectors affected by measures taken to stem the spread of SARS-CoV-2.

Among the limitations of our study, we should mention that we recruited HHs among women who were already participating in the aforementioned clinical trial, and they may not be representative of all HHs in the Balearic Islands’ workforce. However, we believe that our results reflect the main problems of HHs in our setting. On the other hand, we are aware that psychological distress of HHs in other regions might differ. In particular, the pandemic might even have hit HHs harder in countries without programs similar to the Spanish Temporary Workforce Adjustment Plan. Additionally, HHs in the Balearic Islands are hired directly by the different establishments. In contrast, in other regions, outsourcing is the norm, leaving HHs with an even more precarious employment status (i.e., contracts per days and lower salaries). These contextual differences may affect the external validity of our results.

## Data Availability Statement

The data presented in this study are available upon reasonable request from the corresponding author.

## Ethics Statement

The studies involving human participants were reviewed and approved by Balearic Islands Research Ethics Committee (IB 4187/20 PI). The patients/participants provided their written informed consent to participate in this study.

## Author Contributions

XC-A, OB, and JL: conceptualization. XC-A and AL: methodology. XC-A, AL, and LG-A: validation and formal analysis. XC-A and MV-T: investigation. XC-A and JL: writing—original draft preparation. OB, MV-T, LG-A, JL, and AL: writing—review and editing. JL and XC-A: project administration. All authors have read and agreed to the published version of the manuscript.

## Funding

This research is part of a wider Project, “Hotel housekeepers and health,” which is funded by Sustainable Tourism’s Tax Fund (Balearic Islands Government), grant number ITS’17–096.

## Conflict of Interest

The authors declare that the research was conducted in the absence of any commercial or financial relationships that could be construed as a potential conflict of interest.

## Publisher’s Note

All claims expressed in this article are solely those of the authors and do not necessarily represent those of their affiliated organizations, or those of the publisher, the editors and the reviewers. Any product that may be evaluated in this article, or claim that may be made by its manufacturer, is not guaranteed or endorsed by the publisher.

## Footnotes

^1^ http://www.isrctn.com/ISRCTN14664526

^2^ http://www.isrctn.com/ISRCTN14664526
